# Cofactor binding triggers rapid conformational remodelling of the active site of a methyltransferase ribozyme

**DOI:** 10.1016/j.jbc.2024.107863

**Published:** 2024-10-05

**Authors:** Hengyi Jiang, Getong Liu, Yanqing Gao, Jianhua Gan, Dongrong Chen, Alastair I.H. Murchie

**Affiliations:** 1 Shanghai Pudong Hospital, Fudan University Pudong Medical Center, Shanghai, China and Institutes of Biomedical Sciences, Shanghai Medical College, Key Laboratory of Medical Epigenetics and Metabolism, Fudan University, Shanghai, China; 2 Key Laboratory of Metabolism and Molecular Medicine, Ministry of Education, School of Basic Medical Sciences, Fudan University, Shanghai, China; 3 Department of Physiology and Biophysics, Shanghai Public Health Clinical Center, State Key Laboratory of Genetic Engineering, Collaborative Innovation Center of Genetics and Development, School of Life Sciences, Fudan University, Shanghai, China

**Keywords:** enzyme catalysis, methyltransferase ribozyme, ribozymes, RNA recognition, RNA cofactor recognition

## Abstract

The methyltransferase ribozyme SMRZ-1 utilizes S-adenosyl-methionine (SAM) and Cu (II) ions to methylate RNA. A comparison of the SAM-bound and unbound RNA structures has shown a conformational change in the RNA. However, the contribution of specific interactions and the role of a pseudo-triplex motif in the catalytic center on the methylation reaction is not completely understood. In this study, we have used atomic substitutions and mutational analysis to investigate the reaction specificity and the key interactions required for catalysis. Substitution of the fluorescent nucleotide 2-aminopurine within the active ribozyme enabled the conformational dynamics of the RNA upon co-factor binding to be explored using fluorescence spectroscopy. We show that fast co-factor binding (t_1/2_ ∼ 0.7 s) drives a conformational change in the RNA to facilitate methyl group transfer. The importance of stacking interactions at the pseudo-triplex motif and chelation of the Cu (II) ion were shown to be essential for SAM binding.

RNA methylation reactions contribute to a range of cellular functions. Methyl groups are normally transferred from the methyl donor cofactor S-Adenosyl methionine (SAM) to their substrates by protein methyltransferase enzymes. Methylation can happen at carbon, nitrogen, and oxygen atoms within the nucleotide and can change the charge, base-pairing partner, secondary structure stability, and potential for RNA-protein interactions (1). Transfer-RNA and rRNAs are extensively methylated, the modifications can affect the stability of inter and intra-molecular interactions within the translational machinery and appear to be required for accurate translation (1, 2, 3, 4). Transcribed pre-mRNA and mature mRNAs are also methylated and the methylation and demethylation of N^6^-methyladenosine (m^6^A) and N^1^-methyladenosine (m^1^A) have important roles in gene regulation (1, 5, 6). Eukaryotic mRNAs and micro RNAs contain N^7^-methylguanosine (m^7^G) and the m^7^G 5′ cap is important for translation, splicing, and mRNA export (7, 8, 9). The protein methyltransferase enzymes contain a characteristic nucleotide binding “Rossman” fold that binds SAM (10). The sulphonium cation of SAM is highly reactive and methyl-group transfer to nitrogen, oxygen, and sulfur nucleophiles occurs directly in enzyme-SAM-substrate complexes primarily by an SN2 mechanism, although other free radical-based mechanisms of methyl transfer have also emerged (11, 12).

Natural ribozymes play important roles in biological processes; in the translational machinery, a ribosomal ribozyme catalyzes peptide bond formation (13, 14, 15, 16, 17, 18), reviewed in (19). Ribozymes exploit phosphoryl transfer reactions for the maturation of precursor tRNAs by RNase P, intron excision, splicing, and the processing of retroviral and related RNAs (20, 21, 22, 23, 24, 25). Ribozymes have been expanded through *in vitro* selection, and have been engineered to catalyze a range of chemical and basic metabolic reactions to mimic the primitive chemistry of the proposed RNA world (26, 27, 28).

Recently, ribozymes have been identified that transfer methyl groups from m^6^G and O^6^-methyl pre-queuosine ligands to generate m^1^A and m^3^C, respectively, at specific positions in the RNA (29, 30). Using *in vitro* selection methodology, we identified one 33-nt methyltransferase ribozyme that uses SAM as a cofactor (31). The ribozyme is named SMRZ-1 and can catalyze m^7^G modification at a specific position in the RNA. The ribozyme requires Cu (II) ions and functions over a broad pH and temperature range (optimally 60 °C), under optimum conditions the ribozyme methylates at a rate of ∼0.2 per second and has an affinity for SAM (K_M_) of ∼30 μM, which can be considered to be within the physiological range. Bioinformatics searches showed that SMRZ-1 ribozyme motif was widely distributed in various organisms, and some candidate sequences were shown to be active *in vitro*. Potential SMRZ-1 ribozyme sequences were identified in organisms ranging from mammals to simple prokaryotes, including thermophilic strains that grow at high temperatures (31).

The crystal structure and simple mutational analysis revealed that the active site of the ribozyme consisted of 13 core nucleotides. The Ribozyme folds into a novel hairpin structure, in the absence of SAM, it contains an unusual “accordion triple” pseudo triplex RNA fold 5′ to the substrate G nucleotide. In the pseudo triplex, 6 nucleotides (3 from each strand) are accommodated in the space of 2 stacked base pairs as 2 G-C-A (G12-C25-A10 and G26-C11-A24) triples (Fig. 1*D*). The substrate guanine is held in place by stacking interactions with the neighboring 5′G of the accordion triple and the neighboring 3′G. During crystallization, the methyl group is lost, but the position of SAM can be inferred from the resultant SAH bound in the structure. SAM interacts with three regions of the SMRZ-1 ribozyme (Fig. 1, *A*–*C*). SAM binding leads to a dramatic conformational change in the ribozyme, such that the Adenine (A24) in the triple close to the substrate G is displaced by the Adenine of the SAM molecule, which orients the donor methyl group of the SAM towards the N^7^ position of the substrate G for methyl transfer (Fig. 1, *A* and *B*). The bound SAM adopts an elongated conformation in the widened major groove of the SMRZ-1 with the amino acid moiety of SAM bound to the Cu (II) ion (31).

**Figure 1:**
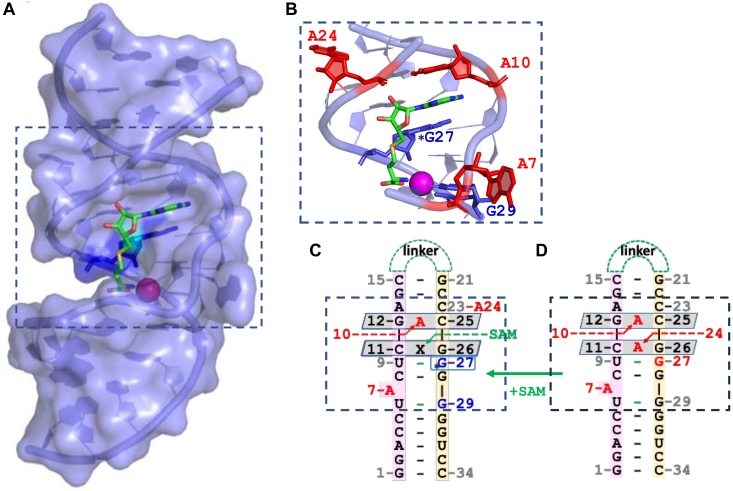
**The active site of the SMRZ-1 methyltransferase ribozyme.***A*, surface depiction of the SAH bound SMRZ-1 ribozyme (taken from PDB accession code 7DWH (31)). Bound SAH and Cu (II) are shown as sticks and spheres respectively, the substrate base G27 is colored in *blue* with N7 picked out in *cyan*. The active site of the ribozyme is boxed. *B*, the active site of the ribozyme (*boxed*), the substrate base G27 (*blue*) is marked with an *asterisk*, and nucleotides A7, A10, and A24 (*red*), and G29 (*blue*) are highlighted. *C*, secondary structure representation of the SAH-bound SMRZ-1 ribozyme, the substrate base ∗G27 is boxed in *blue*, and the *gray* parallelograms represent the C11/SAH/G26 and G12/A10/C25 accordion pseudo triples. SAM binding induces a conformational change in the RNA, note the displacement of A24, compared to (*D*). Secondary structure representation of the SMRZ-1 ribozyme in the absence of SAH, the *gray* parallelograms represent the C11/A24/G26 and G12/A10/C25 accordion pseudo triples. The G12/A10/C25 triple is unchanged upon SAM binding.

In this study, a series of single, and double mutations and atomic substitutions were used to identify the key interactions and requirements for SAM binding and methylation. The incorporation of a fluorescent nucleotide into an active ribozyme allowed changes in the conformation of the RNA upon co-factor binding to be investigated using fluorescence spectroscopy. These results provide fresh insights into the strategies the SMRZ-1 ribozyme utilizes to catalyze methyl-group transfer.

## Results

### The methylation of the N^7^ position of G27 is specific

**Figure 2:**
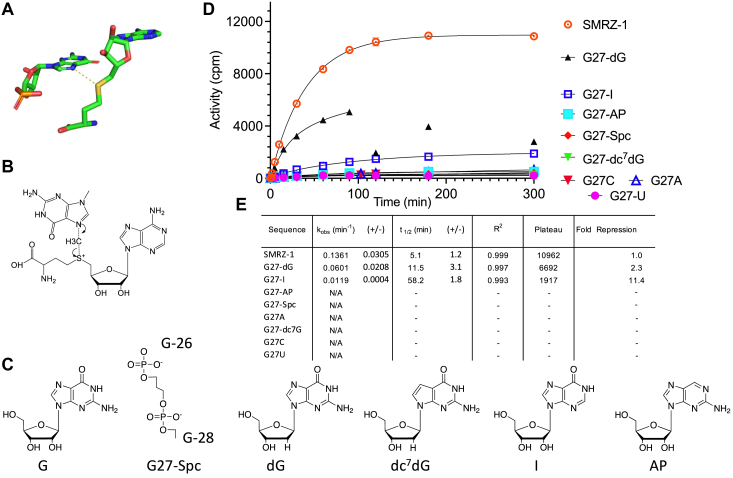
**Atomic substitutions of G27 and their impact on the catalytic activity of SMRZ-1.***A*, the *dotted line* shows the close (4.4 Å) interaction between the N7 of G27 and the sulphur of SAH observed in the crystal structure. *B*, the SN2 mechanism of methyl transfer; nucleophilic attack by the N7 on G27 on the methionine component of SAM. *C*, the chemical structures of the modified nucleotides and spacer substitutions at G27 (G27-Spc) and substitution of deoxy (dG), deaza guanine (dc7G), inosine (I) and 2-amino purine (AP) for G27. *D*, comparison of the catalytic activities (cpm) of SMRZ-1 and RNAs with mutation or modification at G27. Data points are the mean of 2 to 4 independent experiments, and error bars where visible are the standard deviations for each time-point. Note that instability of G27-dm7G is evident for time points >90 min. *E*, the measured rates, kinetic parameters and fold repression relative to SMRZ-1 for the mutated and modified RNAs, the limits (+/−) are the standard deviations for k_obs_ and t_1/2_ from *D*. The data were obtained by fitting to a one-phase or two-phase (SMRZ-1 and G27-dG) exponential model in GraphPad 9.0 as previously described (31), note that the modifications (G27-AP, G27-Spc and G27-dc7G) and the mutations (G27A, G27C and G27U) showed only low levels of H3 incorporation and were considered to be not active (N/A).

Guanosine 27 has been identified as the methylation site of SMRZ-1 by chemical probing. In the crystal structure the N^7^ position of G27 is ∼4.0 Å from the sulfur group of SAH, this close proximity is consistent with the direct transfer of the methyl group to the N^7^ position of G27 through an SN2 mechanism (Fig. 2, *A* and *B*) (31). Mutations of G27 to other nucleotides (G27A, G27U, and G27C) lead to low levels of H^3^ incorporation consistent with background modification of the RNA and are effectively inactive (Fig. 2, *D* and *E*). To examine the specificity of the reaction and the contribution of the specific components of the G27 nucleoside to methylation, the SMRZ-1 ribozyme was chemically synthesized incorporating a series of atomic substitutions and their rates of methylation examined under optimum conditions (60 °C). Substitution of G27 with a deaza dG (dc^7^G, which has a carbon atom at the 7-position) (Fig. 2*C*) results in low levels of H^3^ incorporation, similar to those observed for G27A, G27U, and G27C mutations and can be considered to be inactive (Fig. 2, *D* and *E*). Guanosine 27 is flanked by 5′ and 3′ guanosines (G26 and G28), substitution of G27 by a 3-carbon linker without the guanine base also leads to a loss of activity. These results suggest that methylation is specific for the N^7^ position of G27 and the presence of the guanine base at position 27 is required for methylation. Probably, due to the thermal degradation of m^7^dG, the deoxyribose replacement (G27-dG) (Fig. 2, *C*–*E*) shows a 2-fold reduction in the rate of methylation over the first 90 min; and longer incubations result in a loss of methylation signal. Interestingly, substitution of Inosine at G27 (removal of the 2-amino group of G) results in an 11-fold reduction in rate, while substitution of 2-amino purine (2-AP, which lacks the Oxygen atom at the 6-position) results in a loss of activity (Fig. 2, *C*–*E*), suggesting that the 6-keto group and 2-amino groups both contribute to the activation of the N^7^ position of G.

### An A10 N^6^-A24 phosphate hydrogen bond is essential for SAM binding

**Figure 3:**
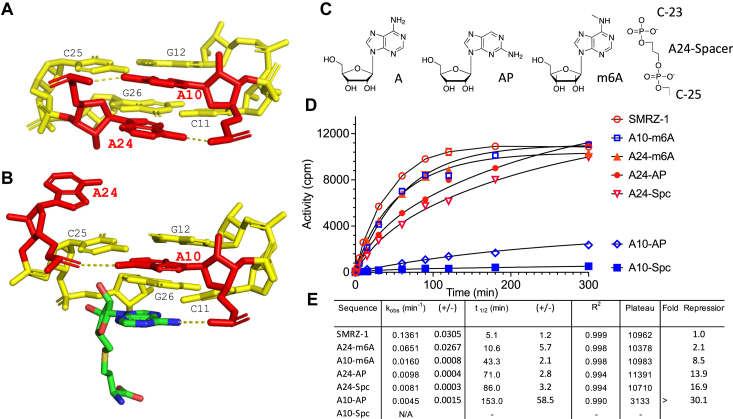
**Investigation of the functional importance of pseudo-triplex forming nucleotides A10 and A24 of SMRZ-1.***A*, the coplanar conformation of the nucleotides of the accordion triple A10, G12-C25 and A24, C11-G26 in the unbound form of the ribozyme (from PDB 7DWH and 7DLZ (31)). The Watson-Crick (WC) GC base pairs are shown in *yellow* and the bases of the pseudo-triplex forming A10 and A24 in *red*, the reciprocal hydrogen bonds between A10 N^6^ and A24 PO2′, and A24 N^6^ and A10 PO2′ are shown as *dashed lines*. *B*, the displacement of A24 upon SAM binding. The W-C GC base pairs are shown in *yellow* and are relatively undisturbed, A10 and A24 are shown in *red*. Note the hydrogen bond between the N^6^ of SAM and the A10 PO2′. *C*, the atomic substitutions made to A10 and A24 and the spacer substitution of A24. *D*, rate measurements of the activity (cpm) of SMRZ-1 RNAs mutated and modified at A10 and A24 compared to the SMRZ-1 ribozyme. Data points are the mean of 3 to 5 independent experiments, error bars where visible are the standard deviations for each time-point. *E*, the measured rates, kinetic parameters and fold repression relative to SMRZ-1 for the mutated and modified RNAs, the limits (+/−) are the standard deviations for k_obs_ and t_1/2_, calculated as for Figure 2*E*, note that the modification (A10-Spc) showed only low levels of H^3^ incorporation and was considered to be not active (N/A).

In the absence of SAM, the SAM binding site of SMRZ-1 ribozyme adopts a novel accordion triple RNA fold in which two Adenosines (A10 and A24) are accommodated into the major groove of the RNA in a pseudo-triple arrangement. The two adenosines are stabilized by stacking interactions and a pair of reciprocal hydrogen bonds (between A10 N^6^ and A24 PO2′ and between A24 N^6^ and A10 PO2′), there are no close interactions with the neighboring co-planar Watson-Crick base pairs (Fig. 3*A*). Upon SAM binding A24 is displaced into the minor groove and its position is occupied by the Adenosine of SAM such that the N^6^ amine group of SAM hydrogen bonds with the PO2′ group of A10 (Fig. 3*B*) (31).

To investigate the contribution of the A10 and A24 hydrogen bonds to SAM binding, atomic substitutions to SMRZ-1 RNA that disrupt or retain the hydrogen bonds were made and their methylation rates were measured. Substitution of 2-aminopurine into the SMRZ-1 RNA at the A10 position (A10-AP), with consequent loss of the N^6^ amine would disrupt the A10 N^6^-A24 PO2′ hydrogen bond and results in a 30-fold reduction in methyltransferase rate. In addition, the excision of A10 (A10-spc) leads to a loss of activity (Fig. 3, *C*–*E*), suggesting that the hydrogen bond that stabilizes A10 is important for SAM binding. Substitution of a methyl-group at N^6^ of A10 (A10-m^6^A) reduces the rate of methylation by 9-fold; mono-methylation of N^6^ appears to weaken hydrogen bonding to A24 PO2′ (Fig. 3, *C*–*E*). These results are in good agreement with the original observation that any mutation to A10 (A10U, A10C, and A10G) is inactive (31), emphasizing the importance of A10 for SAM binding and methylation. However, in contrast to A10-AP mutation, the substitution of 2-aminopurine at A24 (A24-AP) disrupts the hydrogen bond between A24 N^6^ and A10 PO2′ reduces methylation rates 14-fold, although, over the course of the experiment high levels of methylation were achieved. Indeed, the excision of A24 through the substitution of a 3-carbon spacer between C-23 and C-25 results in a 17-fold rate reduction, with high levels of methylation over the course of the experiment (Fig. 3, *C*–*E*). The hydrogen bond (A24 N^6^-A10 PO2′) at position 24 is less critical for SAM binding and methylation, which makes it easier for SAM to displace A24 (Fig. 3*B*). The atomic mutation A24-m^6^A that retains the A24 N^6^-A10 PO2′ hydrogen bond shows a 2-fold reduction in rate. Both atomic mutations (A24-AP and A24-m^6^A) retain methyltransferase activity irrespective of the presence of the A24 N^6^-A10 PO2′ hydrogen bond, further suggesting that the A24 N^6^-A10 PO2′ hydrogen bond is less essential for activity.

### A fast conformational change accompanies SAM binding

The RNAs with the 2-aminopurine nucleobase substitution (A10-AP, A7-AP, and A24-AP) are fluorescent and the fluorescence is sensitive to the local environment of the base within the RNA (32, 33). Changes in fluorescence intensity in the absence or presence of SAM and Cu (II) ions may be used to reveal information on conformational changes upon RNA folding and ligand binding. In the absence of SAM, A7-AP showed a high fluorescence signal presumably due to the extra-helical conformation of A7 in the structure (Figs. 1, *B* and *C* and 6*A*) while the fluorescence of A10-AP and A24-AP RNA is relatively quenched (Fig. S2). On titration of SAM the fluorescence signal of A10-AP is reduced (Fig. S3). As observed in the crystal structures, A24 of the SMRZ-1 ribozyme adopts a different conformation in the presence of SAM (Fig. 3, *A* and *B*). Despite this A24-AP remains active in methylation reactions (Fig. 3, *D* and *E*).

**Figure 4:**
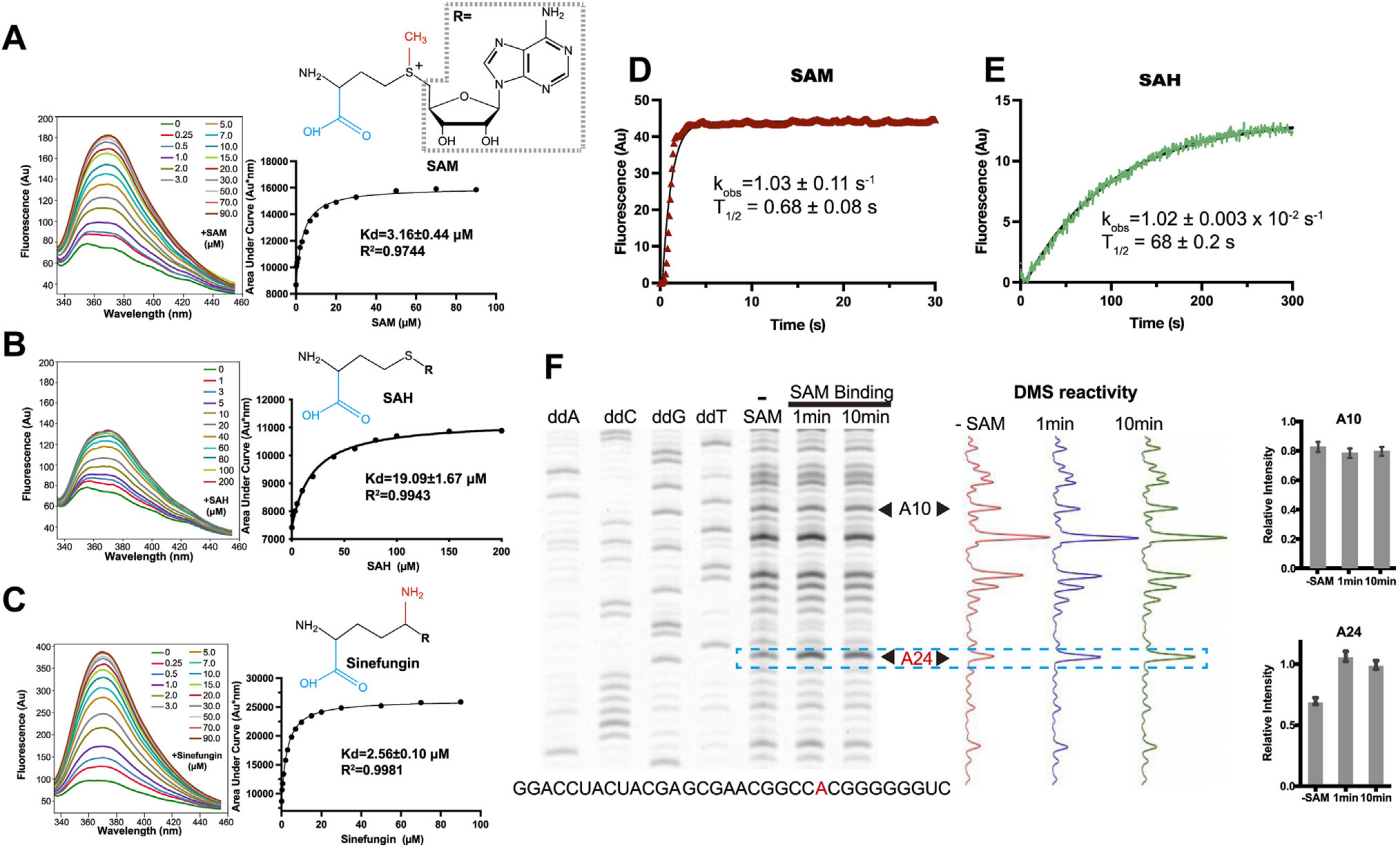
**Investigation of SAM analog binding to SMRZ-1 by fluorescence spectroscopy and chemical probing.***A*, fluorescence emission spectra (λ_ex_ = 315 nm, λ_em_ = 340, 420 nm) of A24-AP on titration of SAM (inset structure), the Kd from the titration is shown in the adjacent panel. *B*, fluorescence emission spectra and Kd of SAH (inset). *C*, fluorescence emission spectra and Kd of Sinefungin (inset). *D*, kinetics of SAM binding to A24-AP with calculated rate constant. *E*, kinetics of SAH binding to A24-AP with calculated rate constant. *F*, chemical probing of SMRZ-1 with DMS, SMRZ-1 was incubated with SAM for 0, 1, and 10 min, after incubation with DMS a SAM-dependent increase in DMS reactivity at A24 relative to A10 was observed.

The fluorescence signal of A24-AP was therefore measured on titration of SAM and the SAM analogs SAH and Sinefungin under standard methylation conditions (with Cu (II)) at 25 °C (Fig. 4, *A*–*C*). We observed a progressive increase in fluorescence signal on titration of SAM with an affinity of 3 μM for A24-AP (Fig. 4*A*). Similar changes also show that the methylation product SAH binds to the RNA with a lower affinity (19 μM) (Fig. 4*B*), than the methyltransferase inhibitor Sinefungin (2.6 μM) (Fig. 4*C*).

The increase in fluorescence in the presence of SAM is consistent with a conformational change at A24. The similar affinities between SAM and Sinefungin suggest that Sinefungin competes directly with SAM in the active site of the ribozyme. To further investigate SAM-induced conformational changes, the SMRZ-1 RNA was subjected to chemical probing with DMS in the absence or presence of SAM and analyzed by gel electrophoresis. An increase in signal at A24 can be seen within 1 to 10 min of the addition of SAM confirming the conformational change at A24 while A10 remains unchanged (Fig. 4*F*).

The changes in fluorescence signal for A24-AP enable ligand binding kinetics for SAM and SAH binding to be measured. In the presence of Cu (II) ions, changes in the fluorescence signal of A24-AP show SAH binding over 300 s with (t_1/2_ = 68 s) and (k_obs_ ∼ 0.010/s) (Fig. 4*E*). In contrast, under the same conditions, SAM binding was complete within ∼1.8 s (t_1/2_ = 0.68 s) (k_obs_ ∼ 1.03/s), which is 100 times faster than SAH binding (Fig. 4*D*). SAH and SAM are distinguished by the presence of the methylsulfonium cation centre of SAM, suggesting that the rapid binding of SAM is driven by electrostatic interactions between SAM and the RNA (Figs. 4*D* and S4).

### Stacking interactions between A10 and the neighboring nucleotides contribute to SAM binding

**Figure 5:**
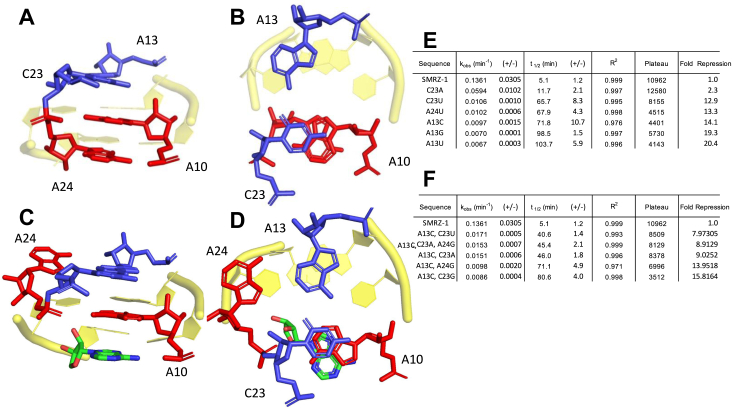
**Analysis of nucleotide substitutions neighboring the A10 pseudo triple.***A*, the conformation of the neighboring A13-C23 bases (*blue*) relative to A10 and A24 (*red*) of the accordion triple in the unbound SAM structure. *B*, *top view* of A13-C23, note stacking of C23-A10-A24. *C*, the conformational change to A24 upon SAM binding, only the Adenosine portion SAM is shown. *D*, *top view* of the putative secondary binding site of A24 upon SAM binding. A24 is positioned beside the A13-C23 wobble pair, note the stacking of C23-A10-SAM. *E*, the measured rates, kinetic parameters and fold repression relative to SMRZ-1 for the single mutant RNAs, taken from 3 to 4 independent experiments as in Figure 2*E*. *F*, the measured rates, kinetic parameters and fold repression relative to SMRZ-1 for the double and triple mutant RNAs (as described in Fig. 2*E*).

Atomic substitution experiments revealed that the hydrogen bond between A10 N^6^ and A24 PO2′ stabilized A10, which was important for SAM binding and methylation by SMRZ-1. In the crystal structure of the SMRZ-1 ribozyme, A10 is stabilized by both hydrogen bonds (A10-A24) and stacking interactions involving the neighboring non-canonical base pair A13-C23. The wobble base pair A13-C23 is positioned immediately above A10-A24 (Fig. 5, *A* and *B*). In the absence of SAM, A10 is stacked between C23 and A24 (Fig. 5*A*). In the presence of SAM, A10 is stacked between C23 and the adenosine of SAM which is held in place for methylation. To investigate the functional contribution of stacking interactions between A24-A10 and A13-C23 on SAM binding, a series of single, double, or triple mutations to the bases adjacent to A10 (at A13, C23 or A24) were constructed and their rates of methylation were measured. When A13 was mutated to other nucleotides (A13C, A13G or A13U), their rates of methylation were reduced by 14 to 20-fold (Figs. 5*E* and S1*A*), suggesting that A13 plays an important role in the stacking interaction. The rates of methylation for single mutation at C23 (C23A and C23U) is also reduced 2.3 and 13-fold respectively (Figs. 5*E* and S1*A*), position C23 may be able to tolerate mutation better than position A13. However, the double mutants A13C/C23A, A13C/C23U, or A13C/C23G show reductions in rate (Figs. 5*F* and S1*B*). The results of atomic substitution on A24 suggest that A24 is not essential for SMRZ-1 activity, consistent with the observation that the previously described mutants A24G and A24C retain activity (31). However, when A24 is mutated to U (A24U) the rate is reduced (Figs. 5*E* and S1*A*). The reciprocal mutant A13U also displays a reduction in methylation rate (Figs. 5*E* and S1*A*). The formation of an AU or UA base pair at positions 13 and 24 disrupts the A13-C23 base pair and the pseudo triplex leading to a reduced rate of methylation activity. The introduction of GC base pairs at Positions 13 and 24, in the double mutant (A13C/A24G) and triple mutant (A13C/C23A/A24G) also show greatly reduced rates (Figs. 5*F* and S1*B*). Although A24 is not essential for methyltransferase activity, mutations at position 24 disrupt pseudo triplex formation and stacking interactions between C23 and A10, impairing the activity of the ribozyme. These results further emphasize the important contribution of the stacking interaction between C23 and A10 to SAM binding. In addition, these mutant sequences may exist as natural genomic sequences, some of which could be as active as the SMRZ-1.

### Extra-helical conformation of A7 accommodates SAM binding stabilized by G29 chelating a Cu (II) ion

**Figure 6:**
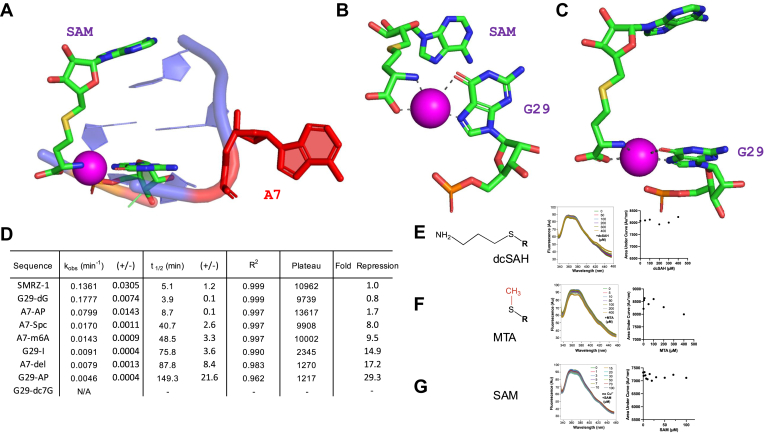
**Investigation of the open major groove of SMRZ-1 and the environment of the methionine group of bound SAM.***A*, the open major groove of SMRZ-1 with SAH bound. The flipped-out base A7 is shown in *red*, the Cu (II) ion is represented by the *magenta sphere*. SAM and G29 are shown in atomic colors. *B*, *bottom view* of the interaction between SAH, Cu (II), and G29. *C*, *side view* of the interaction between SAH, Cu (II) and G29. *D*, rates relative to SMRZ-1 of substitutions to A7 (as 2-amino purine, m^6^A, spacer and deletion as shown in Fig. 5*C*) and G29 (as deoxy G, Inosine, dc^7^G and 2-amino purine, as shown in Fig. 2*C*) as modified SMRZ-1 RNAs taken from 2 to 4 independent experiments as in Figure 2*E*. The measured rates, kinetic parameters and fold repression relative to SMRZ-1 for the modified RNAs (Fig. 2*E*), G29-dG and A7-Ap were fitted to a two-phase exponential model (31), the modification (G29-dc7G) showed low levels of H^3^ incorporation and was considered to be not active (N/A). *E*, fluorescence emission spectra (λ_ex_ = 315 nm, λ_em_ = 340, 420 nm) of A24-AP on titration of dcSAH (R as in Fig. 4*A*), the Kd from the titration is shown in the adjacent panel. *F*, emission spectra on titration with MTA (R as in Fig. 4*A*). *G*, emission spectra on titration of SAM in the absence of Cu (II).

In the SMRZ-1 crystal structure, SAM adopts an extended conformation such that the methionine moiety of SAM is held in place by a single Cu (II) ion that interacts with the amino and carboxy groups of the amino acid and the O^6^ and N^7^ of G29 (Fig. 6, *B* and *C*). In this region of the RNA, A7 is in an unusual extra-helical conformation adjacent to the open major groove that accommodates bound SAM (Fig. 6*A*). To investigate how interactions at A7, G29, and the Cu (II) ion contribute to SAM binding and methylation, a series of atomic substitutions at A7 or G29 were introduced, and their methyltransferase activities were examined.

The substitutions of A7-AP, A7-m^6^A, or a 3-carbon linker without the adenine for A7 retain significant methyltransferase activity (Figs. 6*D* and S5), which is consistent with the initial mutational analysis of A7 showing that mutants A7G and A7U were active (31). However, deletion of A7 (the entire nucleotide) leads to a significant reduction in rate (Figs. 6*D* and S5). Although the nucleoside at position A7 is not required for methylation, the space generated by the nucleotide at this position is essential for SAM binding and ribozyme activity. These results suggest that the extra-helical nucleoside at the A7 position opens up the major groove to provide access for SAM to bind and is essential for methylation. This is also supported by the observation that the mutation A7C, which could form a Watson-Crick base pair with G29, close the major groove, and block SAM binding, is inactive (31). Substitution at G29 with 2-aminopurine at (G29-AP) or deazadG (G29-dc7G) that disrupt Cu (II) ion interactions with the O^6^ or N^7^ of G29 respectively (in the crystal structure), results in a loss of activity, suggesting that O^6^ and N^7^ of G29 are involved in chelating the Cu (II) ion (Figs. 6*D* and S5). Removal of the 2-amino group of G by substitution of inosine at G29 (G29-I) retains low measurable activity suggesting the 2-amino group of G may indirectly contribute to Cu (II) chelation (Figs. 6*D* and S5). The deoxyribose replacement (G29-dG) retains full methylation activity, suggesting that the ribose does not participate in Cu (II) ion chelation (Figs. 6*D* and S5).

The fluorescence signal of A24-AP shows a progressive increase in the titration of SAM and the SAM analogs SAH and Sinefungin under standard methylation conditions which is consistent with ligand-RNA binding (Fig. 4*A*). In contrast, no changes in A24-AP ribozyme RNA fluorescence were detected on titration of the SAM fragments dcSAH that lacks carboxyl the group of SAM, or MTA that lacks the amino acid group (Fig. 6, *E* and *F*). These results support the notion that both the amino acid and carboxyl groups of SAM contribute to coordinating the Cu (II) ion for SAM-RNA binding as seen in the crystal structure (Fig. 6, *B* and *C*). Correspondingly, of the divalent cations, only Cu (II) ions were shown to be essential for SMRZ-1 ribozyme function (31) and in the absence of Cu (II) ions, on titration of SAM, fluorescence measurements of A24-AP indicate no binding (Fig. 6*G*).

Taken together, the results of atomic substitutions at the A7 or G29 position suggest that SMRZ-1 ribozyme methylation requires the extra-helical conformation of A7 to open up the major grove for SAM binding. Concurrently, the O^6^ and N^7^ of G29 and the carboxy and amino groups of the methionine of SAM bind the Cu (II) ion that positions the SAM in place for methylation. These data suggest that the role of the Cu (II) ion in the methylation reaction is not directly catalytic, but that the Cu (II) is involved in positioning the SAM molecule on the RNA.

## Discussion

In order for methyl transfer to take place SAM must bind to the RNA. To accommodate SAM binding, the SMRZ-1 RNA undergoes a relatively fast conformational change: The major groove bordering the substrate G27 nucleotide widens to accept the methionine functional group of SAM and A24 in the pseudo-triple is displaced by the adenosine of SAM. This is in contrast to the DNA methyltransferase enzymes that ‘flip’ the substrate bases out of the B-form helix for methylation by the SAM-bound protein methyltransferase enzyme (10). In the MTR1 ribozyme, the smaller O^6^-methylguanosine cofactor is encapsulated in the active site of the ribozyme close to the adenosine (A63) substrate base. The MTR1 A63 substrate is located at the center of a 3-way helical junction, and may also undergo a conformational change for cofactor binding (30, 34, 35).

Upon SAM binding, A24 was observed to be displaced to a putative secondary binding site in the SMRZ-1 structure. The secondary binding site is positioned on the minor groove side of the distorted A13-C23 wobble pair to make a base triple (Fig. 5, *C* and *D*). There are a number of precedents for the formation of such triples in RNA structures (36, 37). A secondary binding site for A24 would provide additional stability to the ribozyme on SAM binding. The possibility of a binding site for the displaced base is supported by the A24-AP substitution that retains methyltransferase activity and shows only modest changes in fluorescence emission signal upon SAM binding compared to the extra-helical A7-AP signal (Figs. 3, *D* and *E* and 6*D*). Therefore, in addition to stacking interactions between nucleotides in the C23 position and A10, the A13-C23 mis-pair may also contribute to the putative secondary binding site for the nucleotide in the A24 position that is displaced upon SAM binding.

Because the A24 position has a dynamic role and is displaced by SAM to the secondary binding site, it may be possible for some nucleotide substitutions at the A24 position to stack too stably with A10 to be displaced by SAM, such nucleotide substitutions would be expected to reduce the ribozyme activity (*e.g.* A24G mutations) (31). Additionally, substitutions may either bind more stably to the secondary binding site (A13-C23), leading to an increase in activity or may not bind stably to the secondary binding site (A13-C23), leading to a reduction in activity.

After ATP, SAM is the most widely used enzyme cofactor in the cell (38). Most SAM-dependent reactions involve the methylation of the substrate molecule and the generation of SAH by methyltransferase enzymes. The reason that SAM is preferred, compared to other methyl donors is due to the highly exergonic nature of reactions at the charged methylsulfonium center (38). The methylsulfonium center also contributes to the conformational change to the RNA on SAM binding, compared to SAH (that lacks the methylsulfonium center), SAM binds ∼100 times faster (Fig. 5, *D* and *E*). In the presence of SAM the active site of the SMRZ-1 ribozyme is organized such that the lone-pair electrons on the nitrogen nucleophile point toward the incoming methyl group. In this case, the high reactivity of the sulfonium cation enables the direct transfer in an SN2-like mechanism of the methyl group to the nucleophilic nitrogen at N^7^ of the substrate nucleotide, G27 (39, 40) (Fig. 2*B*). SAM binding to A24-AP is significantly faster than the t_1/2_ for methylation of A24-AP (Fig. 3, *D* and *E*) suggesting that further conformational changes to the initial RNA-SAM complex may be required for methyl group transfer.

The original SMRZ-1 ribozyme was refined through truncation to a small active 33-nt ribozyme. However, considerable additional sequence variation is possible (31). Here we have shown that within the constraints for stable SAM binding at A10 through stacking interactions, and the requirement for chelation of a Copper (II) ion at G29, additional sequence variants that have methyltransferase activity have been identified. Indeed, the sequence changes to the original selected ribozyme result in ribozymes with methyltransferase activities that are similar to or exceed the original. This also expands the number of potential natural methyltransferase sequences. The active mutant sequences characterized here were not all included in the original sequence searches and it seems reasonable to predict that further searches would unearth similar natural sequences. The core nucleotides of the ribozyme can be separated into enzyme and substrate strands (31), this modular construction means that enzyme activity may be possible for potential ribozymes with intervening (loop) sequences of various sizes and possible secondary structures that separate enzyme and substrate domains. The natural sequences that have methyltransferase activity *in vitro* display variable loop sequences, although thus far only the activities of relatively short loop sequences have been explored (31).

In conclusion, for the SMRZ-1 ribozyme, the bound SAM cofactor adopts an elongated conformation that interacts with the RNA over three adjacent overlapping sites (Fig. 1), that correspond to the adenosyl, methyl-sulfonium and amino-acid components of SAM. These interactions account for the specificity of the methylation reaction. The factors that contribute to SAM binding and methylation by SMRZ-1 were investigated by using atomic substitutions or mutations. In summary, the extra-helical conformation of A7 opens up the major groove to accommodate SAM binding in the major groove of the RNA (Fig. 6). The adenosine A10, is held in place by stacking interactions with C23 (Fig. 5), and a hydrogen bond to the phosphate of A24 (A10 N^6^-A24 PO2′) (Fig. 3). A10 provides a platform through stacking interactions, and a strategically positioned hydrogen bond acceptor (A10 PO2′) for adenosine binding. Upon SAM binding, the adenosine moiety of SAM stacks with A10, and the carboxy and amino moieties of SAM are held in place in the major groove of the RNA through a bound Cu (II) ion and the Hoogsteen face of G29 (Fig. 6). The binding of SAM to the RNA through these interactions is necessary for methylation to occur, and the combination of the adenosine of SAM and the formation of the SAM-Cu (II)-RNA complex sufficient to displace A24 in the pseudo triple. The methylation site is specific for the N^7^ position of G27 (Fig. 2*D*) Thus, the site of methylation G27 and SAM binding site in SMRZ-1 ribozyme is stabilized and held together by finely-balanced interactions that are essential for SAM binding and methylation.

## Experimental procedures

### Oligonucleotide synthesis

The nucleotide modifications 2-aminopurine, N6-methyl adenosine, deoxyguanine, deoxydeazaguanine, Inosine, and the 3-carbon linker were incorporated by solid-phase oligoribonucleotide synthesis (Takara or Genscript). Unmodified RNA was synthesized by *in vitro* transcription with T7 RNA polymerase.

### Oligonucleotide sequences

All oligonucleotide sequences are provided in the Table S1. The oligoribonucleotide sequences for the atomic mutations are provided in Table S2.

### Reagents

Commercial reagents and suppliers are provided in Table S3.

### Methyltransferase assays

RNA was reacted with SAM in reaction buffer (20 mM Tris-HCl pH 7.4, 20 mM KCl, 1 mM MgCl_2_, 0.1 mM CuSO_4_, 1.0 mM SAM) at 45 °C or the indicated temperature for 2 h. For radioactive assays, 1.275 μM H^3^-labeled SAM (Perkin-Elmer) was included in the buffer. The reaction was quenched with 100 mM EDTA, pH 8.0. The kinetics measurements on SMRZ-1, modified, and mutated RNAs were performed at the optimum temperature for the ribozyme (60 °C), reactions were initiated by the addition of SAM and aliquots for each time point were quenched in 100 mM EDTA as previously described (31). For each data point, the mean of 2 to 5 independent experiments was plotted, and the measured rates and kinetic parameters were extracted by fitting to a one-phase or two-phase exponential model in GraphPad 9.0 as previously described (31), (unprocessed data available; Table S4). The data are shown as time-courses and error bars are the standard deviations for each replicate time-point. The measured rates and kinetic parameters (k_obs_ (min^−1^), t_1/2_ (min), and errors (+/−)), the goodness of fit (R^2^ and Plateau), and fold repression relative to the SMRZ-1 control for comparison are also displayed in tabular form. A competing low-level non-enzymatic chemical methylation reaction by SAM also contributes to background modification of the RNA; samples that displayed low methylation activity under standard reaction conditions over the course of the experiment (300 min) typically having a plateau <6% of the SMRZ-1 value that give poor fitting for rate constant calculations (R^2^ < 0.97) were considered to be not active (N/A).

### Analysis of methyltransferase assays by centrifugal dialysis

The products of methyltransferase assays were filtered through a 3KD centrifugal diafilter. Excess SAM was removed by five successive rounds of washes with MilliQ water and centrifugal diafiltration (3KD, Amicon, Millipore), and the residual modified RNA recovered. Five rounds of dilution and diafiltration were sufficient to remove bound and non-specifically bound SAM. The recovered RNA sample (about 50 μl) was added to 150 μl of scintillant, mixed well, and read with a scintillation counter (Trilux, PerkinElmer).

### Fluorescence spectroscopy

Fluorescence emission spectra of 2-aminopurine (AP) modified SMRZ-1 were measured in an LS50B (PerkinElmer) fluorimeter. Samples were heated at 65 °C for 3 min in buffer 10 mM KCl and cooled to room temperature in a heating block, at 37 °C, the RNA solution was adjusted to a final concentration of 20 mM Tris-HCl pH 7.4, 20 mM KCl, 1 mM MgCl_2_, 0.1 mM CuSO_4_ (when required) the final RNA concentration was 0.5 μM. Samples were incubated in a quartz cuvette with temperature control at 25 °C and fluorescence emission spectra were acquired (λ_ex_ = 315 nm, λ_em_ = 330–460 nm). For binding affinities of SAM and SAM analogs or fragments, aliquots of the small molecules (less than 5% of total RNA volume) were pipetted into a cuvette containing preincubated RNA at 25 °C with mixing and fluorescence emission spectra acquired (λ_ex_ = 315 nm, λ_em_ = 330, 460 nm). Data were fitted to the equation Y = F0 + (Ft − F0) ∗ [Kd + X + R − sqrt (sqr + R + X + Kd) − 4 ∗ X ∗ R]/2/R using Prism 9 (GraphPad), where X is concentration of the small molecule, Y is the area under the emission curve between 330 nm and 460 nm, R is the concentration of RNA and Kd is the binding affinity.

For rate constant measurements of SAH binding, 0.5 μM RNA samples were prepared in buffer (20 mM Tris-HCl pH 7.4, 20 mM KCl, 0.1 mM CuSO_4_, 1 mM MgCl_2_). Prior to the addition of SAH, RNA samples were incubated in cuvettes for 10 min at 25 °C with temperature control, SAH was added and mixed by pipetting. Fluorescence signals were acquired at 0.1 s intervals over 600 s (λ_ex_ = 315 nm, λ_em_ = 370 nm) with slit widths set to 10 nm. SAM binding was measured by stopped-flow fluorescence. Equal volumes of RNA (0.6 μM) and SAM in buffer (20 mM Tris-HCl pH 7.4, 20 mM KCl, 0.3 mM MgCl_2_) were loaded into the injectors of the stopped-flow device (RX2000, Applied Photophysics) and incubated for 10 min at 25 °C prior to mixing. Upon mixing fluorescence signals were acquired at 0.02 s intervals over 400 s (λ_ex_ = 315 nm, (15 nm slit − width), λ_em_ = 370 nm, (10 nm slit − width)). For SAH and SAM fluorescence signals were manually aligned and the processed data fitted to the equation F = A0 + (Plateau − A0) ∗ [1 − exp(−k′ ∗ t)] with Prism 9 (Graphpad), where k′ is the observed rate constant for binding.

### DMS chemical probing

SMRZ-1 RNA transcript RNA (31) was heated to 85 °C for 3 min in 10 mM KCl and cooled from 65 °C to 37 °C. The RNA solution was adjusted to a final buffer composition of 20 mM HEPES, 20 mM KCl, 1 mM MgCl_2_, 0.1 mM CuSO_4,_ and the reaction was initiated by adding SAM to a final concentration of 1.0 mM at 37 °C. After 1 and 10 min 100 μl aliquots were withdrawn and DMS was added to a final concentration of 0.5% with mixing. After 5 min the reactions were stopped with (0.3 M NaAC, 30% (v/v) beta-mercaptoethanol) and ethanol precipitated. The RNA was reverse transcribed (SuperScript III, Thermo) with a FAM-labeled oligonucleotide primer as specified by the manufacturer (31). Sequence markers were generated by reverse transcription of the RNA in the presence of ddNTPs as previously described (31) and samples were electrophoresed in 15% polyacrylamide, 7M Urea gels. Gel images were captured with a Chemi-Doc (Bio-Rad) instrument and quantitated using the integral densitometry software. Fluorescence signals for A10 and A24 were quantitated using Image Lab software (Bio-Rad).

## Data availability

All supporting data are provided within the manuscript, supplementary data and supplementary tables.

## Supporting information

This article contains supporting information.

## Conflict of interest

The authors declare that they have no conflicts of interest with the contents of this article.

## Supporting information

Supplementary Information

Supplementary Table s
