# Supplementary material for: Cofactor binding triggers rapid conformational remodelling of the active site of a methyltransferase ribozyme
Source: J Biol Chem. 2024 Oct 5;300(11):107863. doi: 10.1016/j.jbc.2024.107863 (PMC11566860; doi:10.1016/j.jbc.2024.107863)
Supplement: Supplementary Information [file mmc1.docx]

# **Supplementary Material:**

# Cofactor Binding Triggers Rapid Conformational Remodelling of the Active Site of a Methyltransferase Ribozyme.

Hengyi Jiang ^[a]^, Getong Liu ^[a]^, Yanqing Gao^[b]^, Jianhua Gan ^[b]^

Dongrong Chen*^[a]^ and Alastair I.H. Murchie*^[a]^

Affiliations:

[a] H. Jiang, G. Liu, D. Chen*, A.I.H. Murchie*

Shanghai Pudong Hospital, Fudan University Pudong Medical Center, Pudong, Shanghai 201399, China. And Institute of Biomedical Sciences, Shanghai Medical College, Key Laboratory of Medical Epigenetics and Metabolism, Fudan University, Shanghai 200032, China. Key Laboratory of Metabolism and Molecular Medicine, Ministry of Education, School of Basic Medical Sciences, Fudan University, Shanghai 200032, China.

E-mail: ([ganjhh@fudan.edu.cn](https://mail.google.com/mail/u/0/h/5zybtqj3979i/?&cs=wh&v=b&to=ganjhh@fudan.edu.cn))([drchen@fudan.edu.cn](mailto:drchen@fudan.edu.cn))(AIHM@fudan.edu.cn)

[b] Yanqing Gao^[b]^, Jianhua Gan

Shanghai Public Health Clinical Center, State Key Laboratory of Genetic Engineering, Collaborative Innovation Center of Genetics and Development, Department of Physiology and Biophysics, School of Life Sciences, Fudan University, Shanghai 200438, China

*Correspondence

Key words: Enzyme Catalysis, Methyltransferase Ribozyme, Ribozymes, RNA Recognition

# **Contents**

Title Page Page 1

Authors Page 1

Affiliations Page 1

E-mails Page 1

Contents Page 2

Supplementary Tables Page 3

Supplementary Table 1 Page 3

Supplementary Table 2 Page 3

Supplementary Table 3 Page 4

Supplementary Figure 1 Page 5

Supplementary Figure 2 Page 6

Supplementary Figure 3 Page 7

Supplementary Figure 4 Page 7

Supplementary Figure 5 Page 8

**Supplementary Tables**

**Supplementary Table 1**

**Oligonucleotide Sequences**

All oligonucleotide sequences are provided in the supplementary Table 1.

| Construct | DNA Oligo Sequence |
| --- | --- |
| SMRZ-1 | GACCCCCCGTGGCCGTTCGCTCGTAGTAGGTCCTATAGTGAGTCGTATTA |
| A13G | GACCCCCCGTGGCCGTTCGCCCGTAGTAGGTCCTATAGTGAGTCGTATTA |
| C23U | GACCCCCCGTAGCCGTTCGCTCGTAGTAGGTCCTATAGTGAGTCGTATTA |
| A13C, C23G | GACCCCCCGTCGCCGTTCGCGCGTAGTAGGTCCTATAGTGAGTCGTATTA |
| C23A | GACCCCCCGTTGCCGTTCGCTCGTAGTAGGTCCTATAGTGAGTCGTATTA |
| A13C | GACCCCCCGTGGCCGTTCGCGCGTAGTAGGTCCTATAGTGAGTCGTATTA |
| A13C, C23U | GACCCCCCGTAGCCGTTCGCGCGTAGTAGGTCCTATAGTGAGTCGTATTA |
| A24U | GACCCCCCGAGGCCGTTCGCTCGTAGTAGGTCCTATAGTGAGTCGTATTA |
| A13U | GACCCCCCGTGGCCGTTCGCACGTAGTAGGTCCTATAGTGAGTCGTATTA |
| A13C, A24G | GACCCCCCGCGGCCGTTCGCGCGTAGTAGGTCCTATAGTGAGTCGTATTA |
| A13C, C23A, A24G | GACCCCCCGCTGCCGTTCGCGCGTAGTAGGTCCTATAGTGAGTCGTATTA |
| A13C, C23A | GACCCCCCGGTGCCGTTCGCGCGTAGTAGGTCCTATAGTGAGTCGTATTA |
| T7-forward | TAATACGACTCACTATAGG |

**Supplementary Table 2**

All oligoribonucleotide sequences for the atomic mutations are provided in supplementary Table 2:

| Modified RNAs | RNA Oligo Sequence |
| --- | --- |
| A7-AP | GGGACCU/i2AmPr/CUACGAGCGAACGGCCACGGGGGGUCC |
| A7-m6A | GGACCU/iN6-Me-rA/CUACGAGCGAACGGCCACGGGGGGUC |
| A7-Spc | GGACCU/iSpC3/CUACGAGCGAACGGCCACGGGGGGUC |
| A7-Del | GGACCUCUACGAGCGAACGGCCACGGGGGGUC |
| G29-dG | GGACCUACUACGAGCGAACGGCCACGGG/dG/GGUC |
| G29-I | GGACCUACUACGAGCGAACGGCCACGGG/Inosine/GGUC |
| G29-AP | GGACCUACUACGAGCGAACGGCCACGGG/i2AmPr/GGUC |
| G27-Spc | GGACCUACUACGAGCGAACGGCCACG/iSpC3/GGGGUC |
| G27-dG | GGACCUACUACGAGCGAACGGCCACG /dG/GGGGUC |
| G27-dc7G | GGACCUACUACGAGCGAACGGCCACG/deaza-dG/GGGGUC |
| G27-I | GGACCUACUACGAGCGAACGGCCACG/Inosine/GGGGUC |
| G27-AP | GGACCUACUACGAGCGAACGGCCACG/i2AmPr/GGGGUC |
| A10-AP | GGGACCUACU/i2AmPr/CGAGCGAACGGCCACGGGGGGUCC |
| A10-m6A | GGACCUACU/iN6-Me-rA/CGAGCGAACGGCCACGGGGGGUC |
| A24-AP | GGGACCUACUACGAGCGAACGGCC/i2AmPr/CGGGGGGUCC |
| A24-m6A | GGACCUACUACGAGCGAACGGCC/iN6-Me-rA/CGGGGGGUC |
| A24-Spc | GGACCUACUACGAGCGAACGGCC/iSpC3/CGGGGGGUC |
| G29-dc7G | GGACCUACUACGAGCGAACGGCCACGGG/deaza-dG/GGUC |

**Reagents**

Commercial reagents and suppliers are provided in supplementary Table 3.

**Supplementary Table 3.**

| Commercial Material | Vendor (Cataog ID) |
| --- | --- |
| Phanta Max Super-Fidelity DNA Polymerase Kit | Novozyme (P505-d1) |
| NTP Set 100mM | Thermo (R0481) |
| Rnase inhibitor Ribolock | Thermo (EO0382) |
| DNaseI | Thermo (EN0523) |
| SAM | Sigma (A7007) |
| SAM[3H] | Perkin Elmer (NET155V001MC) |
| Amicon Ultra-0.5 Centrifugal Filter Unit | MilliPore(UFC500324) |
| liquid scintillation cocktails, Ultima Gold | Perkin Elmer (6013321) |
| Millex Syringe Filter | Millipore (SLHV033NK) |
|  |  |

A

B

# **Supplementary Figure 1**

Analysis of nucleotide substitutions neighbouring the A10 pseudo triple.

Supplementary Figure 1A. Substitution analysis of single mutations to the A24 and the A13-C23 pair. Rate measurements of the activity (cpm) relative to SMRZ-1 of the single mutations as described in Figure 2D, rates for the previously described substitutions A24U and A13U (31) are included for reference.

Supplementary Figure 1B. Rate measurements relative to SMRZ-1 of the double and triple mutations (as described in Figure 2D).


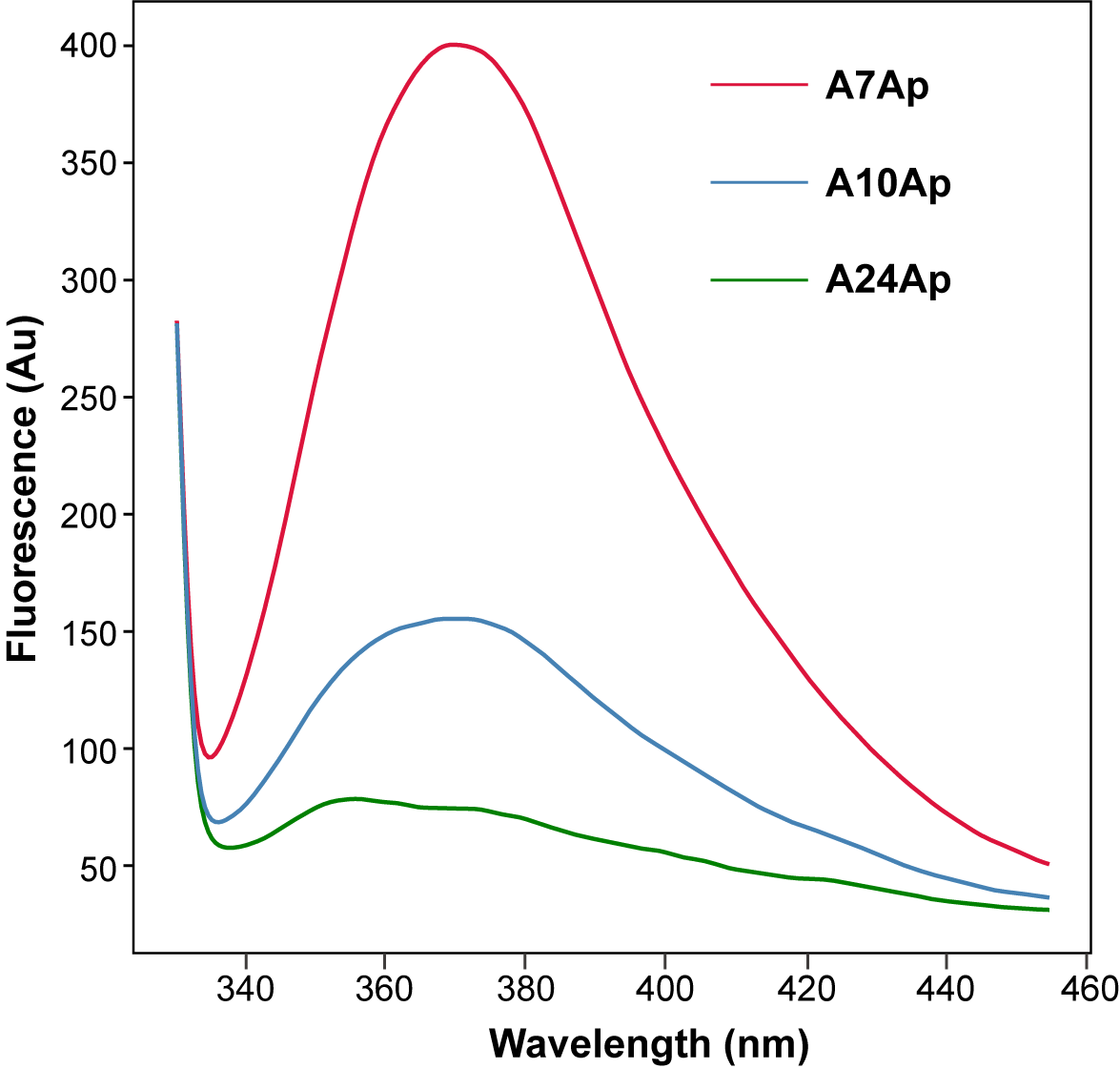


# **Supplementary Figure 2**

Comparison of fluorescence emission spectra (λ_ex_=315 nm, λ_em_= 330 to 440 nm) of A7-AP, A10-AP and A24-AP.


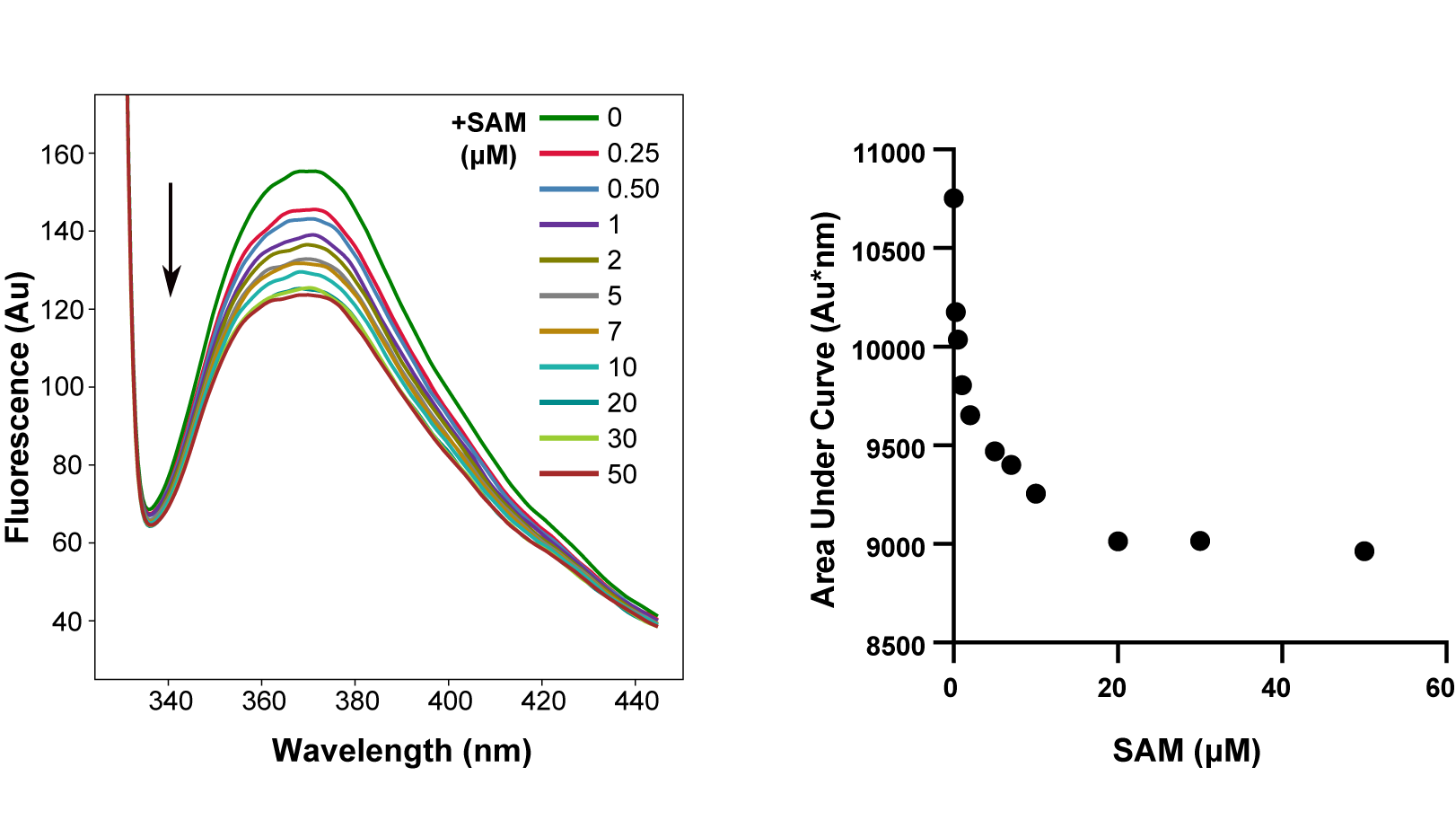


# **Supplementary Figure 3**

Fluorescence emission spectra (λ_ex_=315 nm, λ_em_= 330 to 440 nm) of A10-AP

on titration of SAM.

# **Supplementary Figure 4**

Time courses of SAM binding to A24-AP over 2 independent experiments, error bars (SD) are shown, to clarify visualisation the of data, after 2 seconds the data points are shown for each half second.

# **Supplementary Figure 5**

Rates relative to SMRZ-1 of substitutions to A7 (as 2-amino purine, m^6^A, spacer and deletion as shown in Figure 5C) and G29 (as deoxy G, Inosine, dc^7^G and 2-amino purine, as shown in Figure 2C) as modified SMRZ-1 RNAs as described in Figure 2D.
